# Pancreatic panniculitis: the “bright” side of the moon in solid cancer patients

**DOI:** 10.1186/s12876-017-0727-1

**Published:** 2018-01-04

**Authors:** Elena Guanziroli, Antonella Colombo, Antonella Coggi, Raffaele Gianotti, Angelo Valerio Marzano

**Affiliations:** 1 Dipartimento di Fisiopatologia Medico-Chirurgica e dei Trapianti, Università degli Studi di Milano, Unità Operativa di Dermatologia, I.R.C.C.S. Fondazione Cà Granda, Ospedale Maggiore Policlinico, via Pace 9, 20122 Milan, Italy; 2 Unità Operativa di Dermatologia, I.R.C.C.S. Fondazione Cà Granda, Ospedale Maggiore Policlinico, Milan, Italy

**Keywords:** Pancreatic panniculitis, Pancreatic cancer, Subcutaneous fat necrosis

## Abstract

**Background:**

Pancreatic panniculitis is a rare complication of pancreas disorders occurring in 0.3–3% of patients, most often accompanied by the pancreatic acinar carcinoma.

It presents multiple, painful, deep, ill-defined, red-brown, migratory nodules and plaques of hard elastic consistency; often ulcerated and typically located on the lower proximal and distal extremities.

The pathogenesis is not fully understood, but it is thought to result from lipolysis and fat necrosis with secondary tissue inflammation induced by pancreatic enzymes. Histopathology shows subcutaneous lobular fat necrosis with anuclear adipocytes (called ghost cells) surrounded by a mixed inflammatory infiltrate. Focal calcification may also be seen. The treatment is directed to the underlying disorder, which may result in regression of skin lesions.

**Case presentation:**

We present two cases of pancreatic panniculitis with similar clinical, laboratory, and histopathological features associated with different internal malignancy. The first case, after extensive investigations showed the presence of a pancreatic carcinoma with multiple liver metastases and a poor prognosis. The second one instead is the first case in literature where painful subcutaneous nodules of the legs were the early manifestation of a neuroendocrine carcinoma of the adrenal gland.

**Conclusions:**

Although subcutaneous fat necrosis usually occurs late in the course of a malignancy, recognition of the association with pancreatic panniculitis may prevent a long delay in the diagnosis and management of the occult neoplasm. It should be primarily considered when panniculitis is widespread and persistent, and frequent relapses or tendency to ulcerate of the nodules are regarded as red flags.

## Background

Pancreatic panniculitis (PP) is a rare entity described for the first time by Chiari in 1883 [1], characterized by subcutaneous fat necrosis. It occurs especially in males (M:F = 4:1), with an average age of around 60 and with a higher incidence among alcoholic patients [2]. It is most frequently associated with pancreatic diseases, most commonly acute or chronic pancreatitis and pancreatic carcinoma (usually of acinar cells) [3, 4] and rarely other pancreatic tumors, such as those of neuroendocrine origin [5]. These patients show ill-defined erythematous subcutaneous nodules, more often localized in the lower extremities [6]. The pathogenesis is still unknown, but it is believed to be associated with high levels of serum lipase produced by the neoplasm, causing fat necrosis in tissues [7]. Because the skin lesions often precede the onset of symptoms due to the underlying disease, it is important to consider PP in patients with subcutaneous nodules and elevated levels of pancreatic enzymes, in order to avoid missed or significantly delayed diagnosis [8]. We describe two cases characterized by similar clinical features and an increase of lipase level, with histopathologic features pathognomonic of PP. In both cases skin manifestations were the presenting symptom of an internal malignancy: a pancreatic carcinoma and a neuroendocrine carcinoma of the adrenal gland, which produce high levels of serum lipase responsible for fat necrosis in tissues.

## Case Presentation

### Case 1

**Fig. 1:**
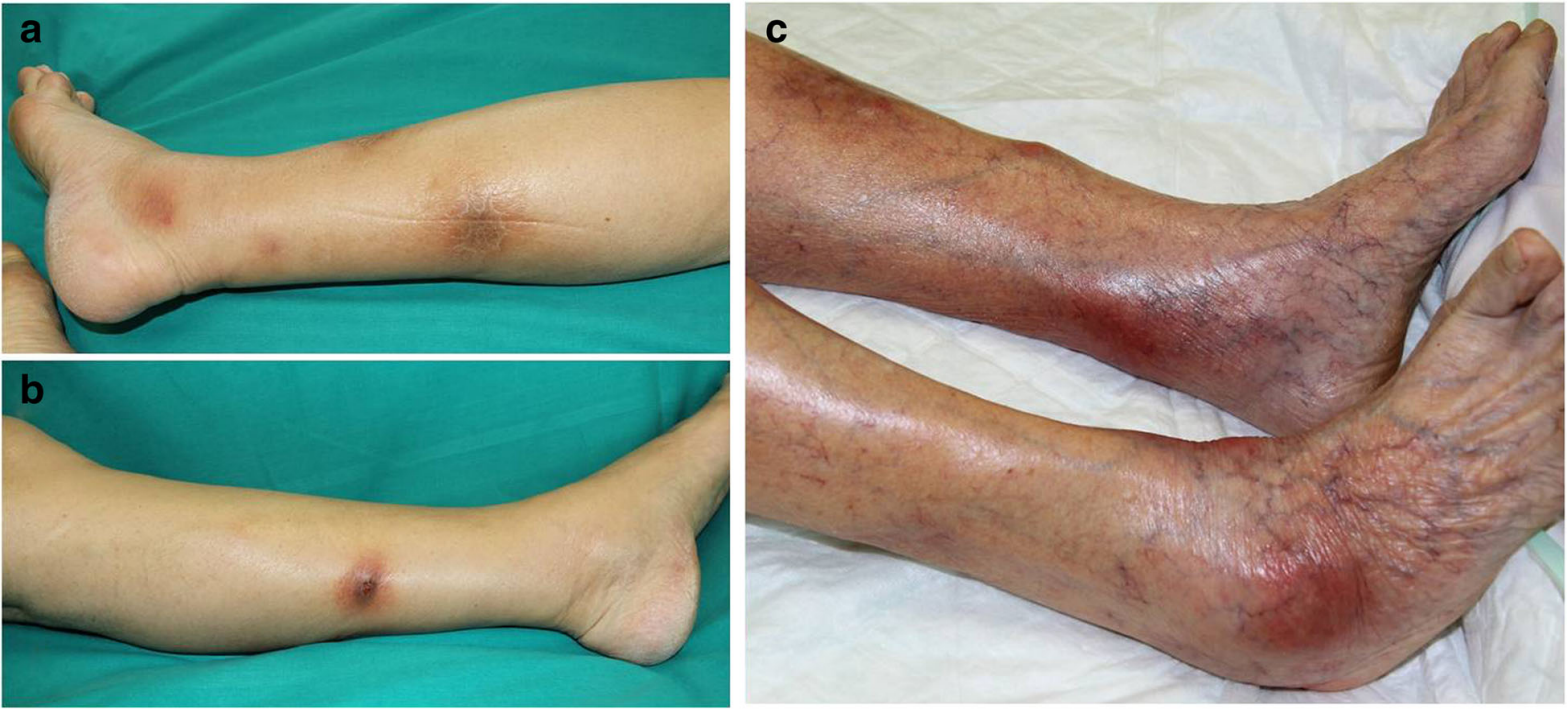
Patient 1: multiple erythematous-brownish subcutaneous nodules on the legs (panel **a**); one of them shows an ulcerated surface (panel **b**). Patient 2: erythematous-violaceous subcutaneous nodules surrounded by a purpuric halo on the lower extremities (panel **c**)

A 77-year-old man presented to our Department with a one-month-history of multiple and slightly painful nodules on his lower legs. His medical history was significant for arterial hypertension, dyslipidemia, and a heart attack and he was in treatment with acetylsalicylic acid, diltiazem, lovastatin and valsartan. Over the following two weeks he noted an increasing number and worsening induration in the nodules; thus, he saw his general practitioner who referred him to a dermatologist in a private practice for an evaluation of the skin lesions. This colleague made a diagnosis of a panniculitis and started a treatment with prednisone 25 mg daily, which was concluded without benefit. On admission, physical examination revealed disseminated, ill-defined, firm, erythematous and violaceous nodules between 2 and 4 cm in diameter (Fig. 1a). A nodule on the medial surface of the left leg presented a superficial pustule and erosion (Fig. 1b). Some of the nodules resolved with painless pigmentation. He was apyrexial with a blood pressure 160/70 mmHg. The remaining physical examination was unremarkable without evidence of organomegaly or lymphoadenopathy. Laboratory investigations showed highly elevated serum lipase levels (6027.2 U/l[13.0–60.0 nv]), with normal amylase, normocytic anemia (Haemoglobin: 9.6 g/dl, MCV 87.9 fl), mild renal impairment (Creatinine: 1.26 mg/dl), increase in inflammatory markers (ESR: 69 mm; CRP: 6.07 mg/dl), elevated GGT (163 mg/dl), and LDH (489 mg/dl) values.

**Fig. 2:**
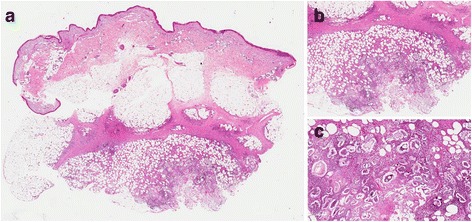
Predominant lobular panniculitis with focal fat necrosis (Panel **a**). Liquefaction phenomena of adipocytes, ghost-like cells and calcium deposits in a predominant neutrofilic inflammatory infiltrate (Panel **b**, **c**)

Tumor markers (CEA, CA 19–9, PSA) were normal, except for the AFP levels (202.7 ng/ml).A biopsy of a subcutaneous nodule showed focal fat necrosis with liquefaction phenomena of adipocytes, ghost-like cells and calcium deposits consistent with PP (Fig. 2). In the suspicion of a paraneoplastic syndrome, the patient, in addition to blood tumor marker investigations, underwent chest radiography (that resulted not significant) as well as abdomen ultrasound, which revealed the presence of multiple focal lesions in the liver (2–7 cm of diameter), and a solid inhomogeneous echostructure, with a necrotic-colliquative central area. A contrast total body computed tomographic (CT) scan confirmed the neoplastic nature of these findings and showed a mass of about 3 × 4 cm in the tail of the pancreas, referable in the first hypothesis to the primary cancer.

The patient was subsequently referred to an oncologist, that in relation to the extension of the disease, administered only supportive symptomatic therapy. His condition severely deteriorated in the meantime and the patient died two months later.

### Case 2

An 85-year-old woman was admitted to our Department with a 6-month history of painful erythematous nodules located bilaterally on the legs, progressively developed and worsened. The first diagnosis, made by an external dermatologist, was erythema nodosum and the patient was placed on systemic antibiotics and steroids therapy with josamicin 2 g/day and prednisone 25 mg/day, leading however a worsening of the skin lesions.

The patient had good general condition and she just took propafenone for previous episodes of ventricular extrasystoles. She did not report any weight loss, fever, nausea or vomiting, abdominal pain or problems with food intake. Her family history was significant for cardiovascular diseases. The physical examination revealed multiple firm erythematous-violaceous nodules on the legs and the ankles; of various sizes, from about 2 to 4 cm in diameter, and painful (Fig. 1c). A deep biopsy taken from a lesion showed a predominantly lobular panniculitis with liquefactive fat necrosis and adipocytes saponification. Calcium deposits were associated with pseudomembranes. Laboratory examinations showed an elevated concentration of serum lipase (3467 U/l) with normal level of amylase. Inflammatory markers were increased (ESR: 63 mm; CRP: 2.43 mg/dl; beta2 microglobulin 3.3 mg/dl), as well as white blood cells (11.02 10 × 3/mmc). All other routine tests and tumor markers (CEA, CA125, CA15.3, CA 19–9, AFP) were normal. A total body CT scan was performed to investigate the lipase increased and it showed a voluminous solid neoplasm of 8 cm in diameter at the level of the left adrenal gland, which was not evident on the abdomen ultrasound, performed 6 months before. Ultrasound-guided needle biopsy of the mass was performed and it revealed a well-differentiated endocrine tumor (with this immunohistochemical profile: Cytokeratin PAN (+), Synaptophysin (+), Chromogranin (−), Cytokeratin 7 (−), Vimentin (−), CD10 (−).

The patient was subsequently sent to an oncology operating unit but she died of acute myocardical infarction while she was waiting for surgery.

## Discussion and Conclusions

The described cases are two different examples of PP. In 40% of cases of PP, skin lesions precede the symptoms of underlying diseases and the interval between cutaneous findings and discovery of abdominal disorders goes from 1 to 7 months, [8, 9] an early and proper diagnosis being thus crucial. PP usually presents with erythematous-violaceus plaques and nodules predominantly located on the legs (around the ankles and pretibial region), buttocks or trunk, which may resolve spontaneously or may ulcerate, draining an oily brown, sterile, viscous substance due to liquefactive fat necrosis. [10, 11]. Clinically, it is often difficult to make a differential diagnosis between the various types of panniculitis: especially erythema nodosum, lupus panniculitis, panniculitides in sarcoidosis, and erythema induratum/nodular vasculitis.

Considering the wide spectrum of differential diagnoses a deep skin biopsy that includes the subcutaneous tissue is mandatory for the diagnosis. Histopathological examination shows lobular fat necrosis with a predominantly neutrophilic inflammatory infiltrate. Focal calcification and anuclear adipocytes, within a thick, shadowy wall (ghost cells) are characteristic [12]. Laboratory investigations usually reveal raised amylase, lipase and trypsin levels and sometimes the presence of elevated tumor marker levels, especially carcinoembryonic antigen [13]. Interestingly, in the first patient, elevated lipase were found even though he had normal amylase. Although the reason of this discrepancy remains unclear, there are other similar cases in literature reporting the same blood changes [12, 14]. Moreover, some cases of PP occurring in patients with normal pancreatic enzymes have been reported. It has been hypothesized that some patients are unable to degrade pancreatic enzymes, possibly due to deficiencies of enzymes like alpha-1 antitrypsin [12]. The pathogenesis of pancreatic panniculitis is not fully understood, but it seems that the release of high amounts of pancreatic enzymes such as trypsin, amylase, lipase and phospholipase may lead to a focal necrosis of lipids and a concomitant inflammatory reaction [6, 15, 16]. Clinicians should be aware that panniculitis may be the warning signal of serious diseases, such as pancreatic cancer or other types of cancer, and may precede the symptoms and signs typically related to these conditions. Therefore, it is crucial to perform laboratory exams and instrumental investigations to disclose the presence of underlying diseases [17].

In the majority of the reported cases, this form of panniculitis is caused by pancreatic diseases [18, 19], the first case we described is a clear example of this. Our second patient is instead a very unique case in which the cause of panniculitis was an endocrine tumor, localized in the adrenal system which produces lipase. This is the first case in literature of a panniculitis associated to an endocrine tumor of the adrenal gland. The production of lipase by this tumor (3467 U/l was the laboratory data of our patient) clearly leads to the same clinical manifestation associated with pancreatic diseases and the pathogenesis could be similar to panniculitis caused by pancreatic cancers. Although subcutaneous fat necrosis generally occurs late in the course of a malignancy, recognition of the association with PP may prevent a long delay in the diagnosis and management of the occult neoplasm. It should be primarily considered when panniculitis is widespread and persistent, and frequent relapses or tendency to ulcerate of the nodules are regarded as red flags.

## Abbreviations

AFP: Alpha fetoprotein
CA 19–9: Carbohydrate antigen 19–9
CA125: Carbohydrate antigen 125
CA15.3: Carbohydrate antigen 15.3
CEA: Carcinoembryonic antigen
CRP: C-reactive protein
CT: Computed tomography
ESR: Erythrocyte sedimentation rate
GGT: Gamma glutamyl transferase
LDH: Lactate dehydrogenase
MCV: Mean cell volume
PP: Pancreatic panniculitis
PSA: Prostatic antigen
